# A specific and ultrasensitive Cas12a/crRNA assay with recombinase polymerase amplification and lateral flow biosensor technology for the rapid detection of *Streptococcus pyogenes*

**DOI:** 10.1128/spectrum.00345-24

**Published:** 2024-09-10

**Authors:** Yu Cheng, Jiawen Lyu, Jiangfeng Han, Long Feng, Xiangmei Li, Pei Li, Shanfeng Zhang, Wenqiao Zang

**Affiliations:** 1 School of Basic Medical Sciences, Zhengzhou University, Zhengzhou, Henan, China; 2 Centre for Student Innovation and Entrepreneurship, School of Basic Medical Sciences, Zhengzhou University, Zhengzhou, Henan, China; 3 Grade 2022, Stomatology Major, Zhengzhou University, Zhengzhou, Henan, China; 4 Medical College, Henan University of Chinese Medicine, Zhengzhou, Henan, China; 5 Department of Clinical Medicine, School of Medicine, Zhengzhou University, Zhengzhou, Henan, China; 6 School of Medicine, Henan University of Chinese Medicine, Zhengzhou, Henan, China; 7 Department of Respiratory Medicine, The First Affiliated Hospital of Zhengzhou University, Zhengzhou, Henan, China; Quest Diagnostics Nichols Institute, Chantilly, Virginia, USA

**Keywords:** *Streptococcus pyogenes*, CRISPR technology, diagnostic methods

## Abstract

The potential of CRISPR/Cas systems for nucleic acid detection in novel biosensing applications is remarkable. The current clinical diagnostic detection of *Streptococcus pyogenes* (*S. pyogenes*) is based on serological identification, culture, and PCR. We report a rapid, simple, and sensitive method for detecting and screening for *S. pyogenes*. This novel method is a promising supplemental test. After 10 min of the sample processing and 10 min of recombinase polymerase amplification, followed by 10 min of Cas12 reaction and 3 min of lateral flow biosensor (LFB) readout, a visible outcome can be observed without the need for magnification within 33 min. This platform is robust, inexpensive, and appropriate for on-site testing. A new technique for detection was created using CRISPR-Cas12a technology, which includes two measurements: a fluorescent-CRISPR-*S. pyogenes* test and a LFB-CRISPR-*S. pyogenes* test. An approach utilizing CRISPR Cas12a was developed, and the accuracy and precision of this technique were assessed. The LoD for the fluorescence-CRISPR- *S. pyogenes* assay was 1 copy/μL, and the technique effectively differentiated *S. pyogenes* from other microorganisms. Moreover, the detection outcomes were presented in a user-friendly manner using lateral flow biosensor strips. Conclusion: A rapid and sensitive Cas12a/crRNA assay using recombinase RPA and LFB was developed to detect *S. pyogenes*. The Cas12a/crRNA-based assay exhibited high specificity among different bacteria strains and extremely high sensitivity. The accuracy and rapidity of this method make it a promising tool for *S. pyogenes* detection and screening.

**IMPORTANCE:**

Patients may experience a range of symptoms due to *Streptococcus pyogenes* infections, including superficial skin infections, pharyngitis, and invasive diseases in subcutaneous tissues like streptococcal toxic shock syndrome. At present, the clinical diagnostic detection of *S. pyogenes* is based on serological identification, culture, and PCR. These detection methods are time-consuming and require sophisticated equipment, making these methods challenging for routine laboratories. Thus, there is a need for a detection platform that is capable of quickly and accurately identifying *S. pyogenes*. In this study, a rapid and sensitive Cas12a/crRNA assay using recombinase RPA and LFB was developed to detect *S. pyogenes*. The Cas12a/crRNA-based assay exhibited high specificity among different bacteria strains and extremely high sensitivity. This method probably plays an important role for *S. pyogenes* detection and screening.

## INTRODUCTION

*Streptococcus pyogenes*, a pathogen that causes β-hemolysis and is gram-positive, exclusively infects humans in their natural environments. While *S. pyogenes* can colonize the throat and skin without causing symptoms, it is also responsible for a diverse range of illnesses (1–4). Patients may experience a range of symptoms due to *S. pyogenes* infections, including superficial skin infections, pharyngitis, and invasive diseases in subcutaneous tissues like streptococcal toxic shock syndrome (5, 6). At present, the clinical diagnostic detection of *S. pyogenes* is based on serological identification, culture, and PCR. These detection methods are time-consuming and require sophisticated equipment, making these methods challenging for routine laboratories. Thus, there is a need for a detection platform that is capable of quickly and accurately identifying *S. pyogenes*.

The CRISPR-Cas system is an acquired defense mechanism that safeguards certain bacteria and archaea against viral and plasmid intrusion by identifying distinct nucleic acid sequences. The CRISPR-Cas system is capable of identifying and cleaving target nucleic acids rapidly, efficiently, and precisely, making this system well-suited for genetic engineering. When combined with existing molecular biotechnology, the CRISPR/Cas-based system can detect specific nucleic acids with attomolar sensitivity ((7)^8^–12).

The Cas12a protein belongs to the second major class of CRISPR/Cas Type V systems. Under the guidance of crRNA, Cas12a can specifically bind and cut exogenous dsDNA. For on-site detection, recombinase polymerase amplification (RPA) is a novel method that works under isothermal conditions (13–15). It is important to have sensitivities, time-savings, labor-savings, and instrumentation-independent detection methods in order to meet all pathogen detection objectives (8, 16). CRISPR-Cas-based detection methods may satisfy these requirements.

The combination of RPA technology and Cas12a was utilized to create DETECTR systems, which are capable of highly sensitive detection of nucleic acids (13, 17–21). The Cas12a-based system exhibits a nonspecific endonuclease activity (trans-cleavage activity) toward the surrounding ssDNA. This endonuclease activity is important when using CRISPR/Cas12a in nucleic acid detection methods, which include crRNA as the characteristic sequence recognition, Cas12a as the effector protein, and RPA as the nucleic acid amplification method. DETECTR with CRISPR/Cas12a can be combined with RPA (isothermal amplification) to achieve rapid amplification under 37°C with very high sensitivity while reducing equipment dependence and detection time.

At point-of-care, a biosensor using gold nanoparticles (AuNPs) on a lateral flow platform can be utilized. A colorimetric change occurs as nanoparticles aggregate in the presence of an analyte, and the signal is visible without complicated instruments. The study utilized CRISPR/Cas12a along with RPA technology and LFB to develop a fast, precise, and sensitive detection method for *S. pyogenes*. This method can be applied to the prevention and treatment of pyogenic infections and allergic diseases.

## MATERIALS AND METHODS

### RPA primer and CRISPR RNA (crRNA) design

The complete sequence of *S. pyogenes* is downloaded from GenBank. Subsequently, we analyzed the conserved specific genes as target genes. The sdaB gene was identified as the target gene. Upstream and downstream primers of RPA were designed for the target segments containing PAM sequences. Subsequently, the conserved specific segments of the sdaB gene containing PAM sites were analyzed as guide sequences of crRNAs. Candidate guide sequences were searched in BLAST to further confirm their conservative specificity.

The primers and crRNA are shown in Table 1. Sangon Biotech (Shanghai, China) synthesized the primers and crRNA. DNA extraction from *S. pyogenes* (ATCC19615) standard strains (5 × 10^6^ CFU/mL) was performed using the Bacterial Genomic DNA Extraction Kit (Bioteke Corporation, Beijing, China) according to the guidelines provided by the manufacturer.

**TABLE 1:** Primer and crRNA sequences for *S. pyogenes*-CRISPR detection

Name	Sequence(5′−3′)
Forward primer	tcaatggtagctcttgtatcagccacaatgg
Reverse primer	AAAGAGTGCTGGAGTAATCTGACTAGTACCT
crRNA1	UAAUUUCUACUAAGUGUAGAUGGUACUUGCUUGCGCCAUCAUUU
crRNA2	UAAUUUCUACUAAGUGUAGAUGAACAACAUCAUUUGAGACCUGU

### RPA amplification

For RPA, the primers utilized were as follows: the forward primer with the sequence 5′-TCAATGGTAGCTCTTGTATCAGCCACAATGG-3′ and the reverse primer with the sequence 5′- AAAGAGTGCTGGAGTAATCTGACTAGTACCT-3′. Table 2 displays the reaction system. The mixture of substances was kept at 37°C for 5, 10, 15, 20, 25, and 30 min. The RPA product was visualized using agarose gel electrophoresis.

**TABLE 2:** RPA reaction system

	Reagent	Volume
1	Reaction buffer (2×）	10 µL
2	Core mix (4×）	5 µL
3	Forward primer (20 µM)	0.5 µL
4	Reverse primer (20 µM)	0.5 µL
5	DNA template	2 µL
6	Starter (10×)	2 µL
A total volume of 20 µL		

### CRISPR Cas12a reaction

Table 3 displays the CRISPR-Cas12a reaction system. The samples were gently mixed and centrifuged (violent oscillations of vortices should be avoided) three times. The reaction tube was then placed in the Fluorescent quantitative PCR instrument (LongGene Scientific Instruments Co., Ltd. Hangzhou, China). At 37°C, the reaction mixture was incubated for 30 min.

**TABLE 3:** CRISPR-Cas12a reaction system

	Reagent	Volume
1	Cleavage buffer (10×）	2 µL
2	Reporter (4 µM）	0.6 µL
3	Cas12a (1 µM）	1 µL
4	crRNA (1 µM)	1 µL
5	RPA product	1 µL
6	Nuclease-free H_2_O	14.4 µL
A total volume of 20 µL		

### Sensitivity of the CRISPR-*S. pyogenes* assay

In order to detect the sensitivity of the CRISPR-*S. pyogenes,* the plasmids containing RPA amplified fragment of sdaB (pUC18-sdaB) were extracted. Series concentration pUC18-sdaB DNA was tested both the fluorescence-CRISPR-*S. pyogenes* and the LFB-CRISPR-*S. pyogenes* assays. The CRISPR systems in the LFB-CRISPR-*S. pyogenes* assay and the fluorescence-CRISPR-*S. pyogenes* assay were similar, but the probes were different. A probe labeled with FAM-TTATT-BHQ1 was used in the fluorescence-CRISPR-*S. pyogenes* test. In the LFB-CRISPR-*S. pyogenes* assay, a dual-labeled FITC and biotin probe were used. The LFB-CRISPR-*S. pyogenes* test involved adding 50 µL of water to the 20 µL CRISPR reaction product, followed by loading the resulting 70 µL mixture onto the LFB and incubating it for 3 min.

### Specificity of the CRISPR- *S. pyogenes* assay

The bacterial strains in Table 4 were used for specificity assay. Genomic DNA was extracted with 5 × 10^6^ CFU/mL standard strains bacteria. Fluorescence-CRISPR assay and LFB- CRISPR assay were then performed after RPA amplification.

**TABLE 4:** The bacterial strains for specificity assay

Number	Strain
Positive	*Streptococcus pyogenes* (ATCC 19615)
N1	*Streptococcus pneumoniae* (ATCC49619)
N2	*Streptococcus mitis* (ATCC6249)
N3	*Streptococcus agalactiae* (ATCC13813)
N4	*Streptococcus dysgalactiae subsp* (ATCC12388)
N5	*Streptococcus anginosus* (ATCC33397)
N6	*Streptococcus bovis* (ATCC35034)
N7	*Streptococcus gallolyticus* (ATCC49147)
N8	*Streptococcus equinus* (JCM7879)
N9	*Staphylococcus aureus* (ATCC25923)
N10	*Staphylococcus epidermidis* (ATCC12228）
N11	*Escherichia coli* (ATCC25922）
N12	*Pseudomonas aeruginosa* (ATCC27853）
N13	*Listeria monocytogenes* (ATCC19118)

### Detection of a clinical DNA sample

Thirty-six conjunctival sac secretions from conjunctivitis patients and 38 throat swab samples from pharyngitis patients were selected as clinical samples. Conjunctival sac secretions from conjunctivitis patients were collected in January 2024. Throat swabs were collected from 38 patients with pharyngitis in July 2024. The studies involving human participants were reviewed and approved by Life Science Ethics Review Committee, Zhengzhou University (NO. ZZUIRB2024-146). The clinical samples were detected with Real-time PCR and LFB-CRISPR-*S. pyogenes* assay.

## RESULTS

### Developing the RPA and CRISPR-*S. pyogenes* systems

**Fig 1:**
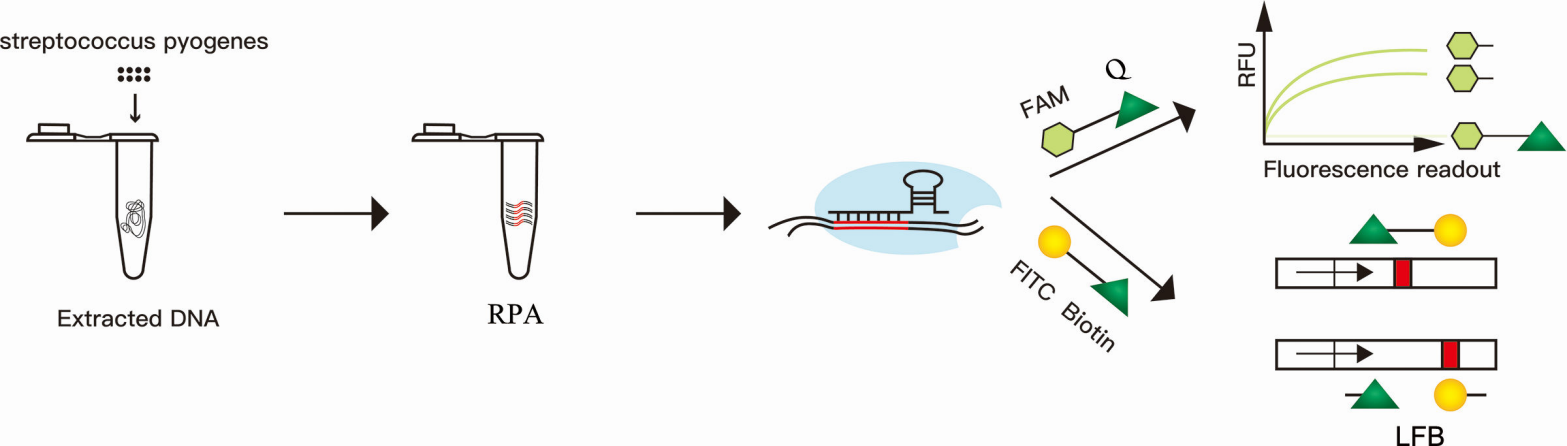
Establishment of the RPA and CRISPR-*S. pyogenes* systems. The new assay consisted of three steps: DNA extraction, RPA, and fluorescence signal detection or LFB.

The accurate identification of *S. pyogenes* DNA was achieved by combining the CRISPR-Cas12a system and RPA. The new CRISPR-RPA assay can be routinely and rapidly performed. The new assay consisted of three steps: DNA extraction, RPA, and fluorescence signal detection or LFB (Fig. 1). RPA was utilized to amplify the segment of the sdaB gene, and the amplicons were subsequently included in the CRISPR-Cas12a reaction system. Upon binding and cleaving the target double-stranded DNA (cis-cleavage), which is complementary to the crRNA, the CRISPR-Cas12a/crRNA complex initiates collateral cleavage activity that non-selectively degrades the single-stranded DNA reporter (trans-cleavage), resulting in the generation of a fluorescent signal or alteration in color. Fluorescence signals can be detected with a fluorescence reader. The color change for the LFB is visible to the naked eye.

### Developing the LFB-CRISPR-*S. pyogenes* systems

The fluorescence-CRISPR-*S. pyogenes* assay, serving as a straightforward method for detecting *S. pyogenes*, still necessitates the use of fluorescence excitation sources and fluorescence readers. The inclusion of LFB technology enabled us to simplify and make the assay more practical, eliminating the need for complex instruments. The LFB-CRISPR-*S. pyogenes* assay can be visually interpreted. The probe is degraded as a result of Cas12a activating the target sequence. The CRISPR systems in the LFB-CRISPR-*S. pyogenes* and fluorescence-CRISPR-*S. pyogenes* assays are similar, but different detection probes are used. The LFB-CRISPR-*S. pyogenes* assay employs a dual-labeled FITC and biotin probe, and the product is loaded onto the LFB.

**Fig 2:**
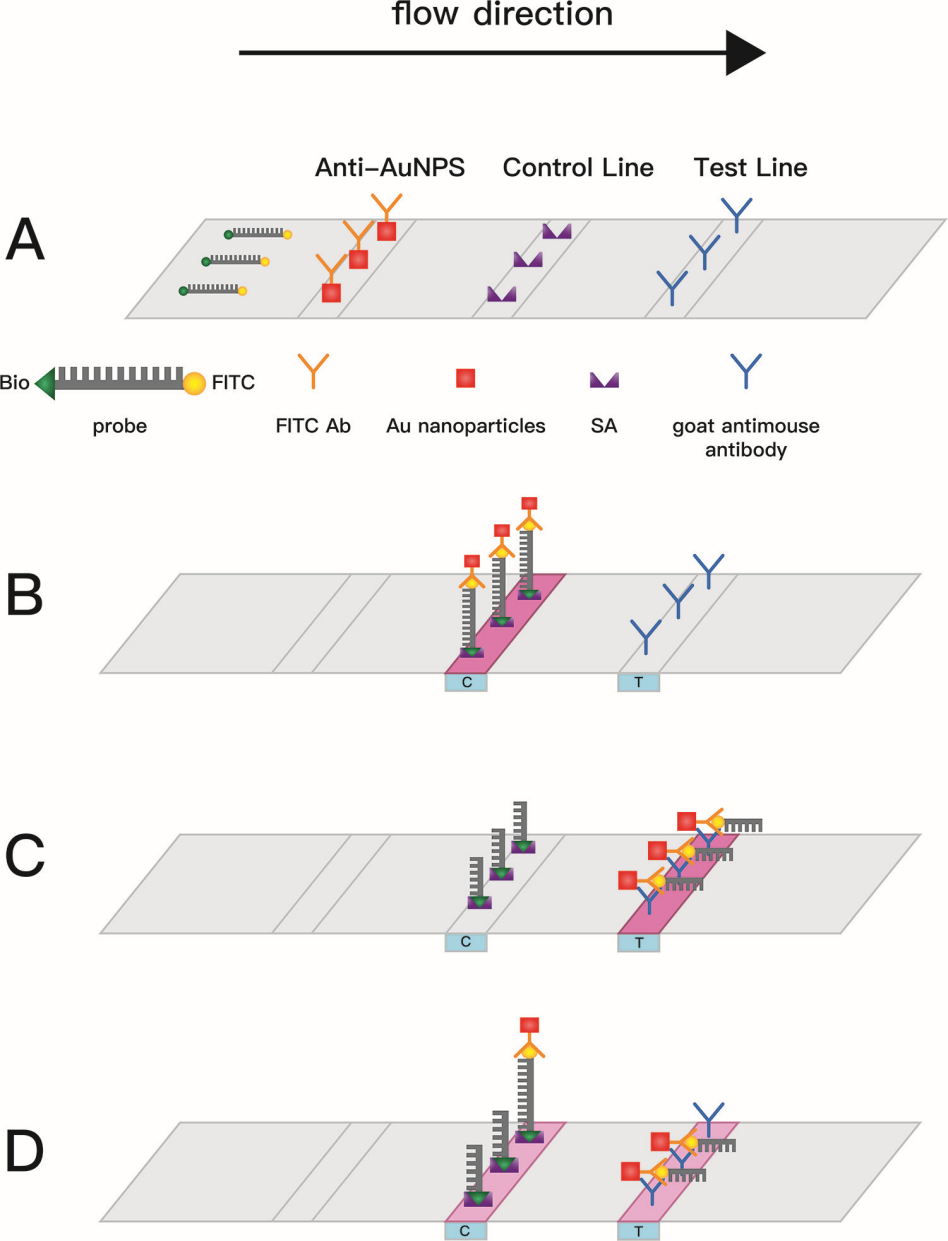
Principle and establishment of LFB-CRISPR-*S. pyogenes* assay. (**A**) The LFB includes gold nanoparticle-labeled anti-FITC antibodies on the sample pad, while the nitrocellulose membrane has two lines with bound streptavidin (SA) and a goat anti-mouse antibody. (**B**) In the absence of target DNA, the probe stays intact and Anti-AuNPs are captured by SA on the control line; consequently, a red line appears on the control line. (**C**) When target DNA is present, the probe is degraded by Cas12a-mediated nonspecific cleavage, and the Anti-AuNPs are captured on the test line. Therefore, a red line appears on the test line. (**D**) When a small amount of target DNA exists, the probe is partially degraded by nonspecific Cas12a cleavage, and some of the Anti-AuNPs are captured on the control line; the other Anti-AuNPs are captured on the test line. Therefore, the red lines appear simultaneously on the control and the test lines.

The LFB on the example pad includes gold nanoparticle-labeled anti-FITC antibodies, while the nitrocellulose membrane (Fig. 2A) features two lines of bound streptavidin (SA) and goat anti-mouse antibodies. If there is no target DNA, the probe stays intact, and Anti-AuNPs are captured by SA on the control line; consequently, a red line appears on the control line (Fig. 2B). In the presence of target DNA, the probe is degraded by Cas12a-mediated nonspecific cleavage, causing the Anti-AuNPs are captured on the test line instead of the control line. Therefore, a red line appears on the test line (Fig. 2C). When a small amount of target DNA exists, the probe is partially degraded by nonspecific Cas12a cleavage, and some of the Anti-AuNPs are captured on the control line; the other Anti-AuNPs are captured on the test line. Therefore, the red lines appear simultaneously on the control and the test lines (Fig. 2D).

### RPA reaction and optimization of RPA detection

**Fig 3:**
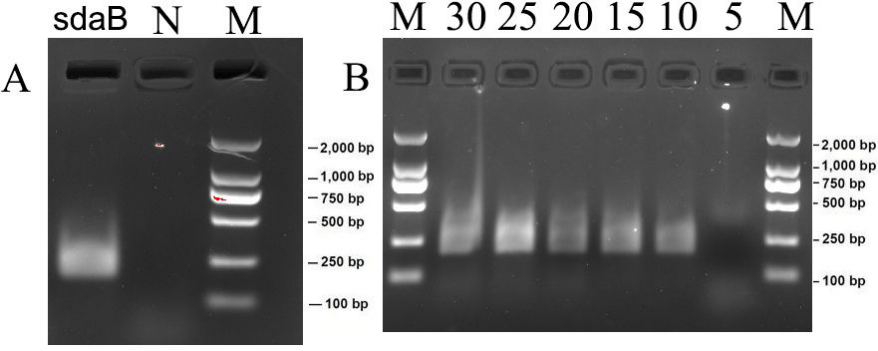
Agarose gel electrophoresis results. (A) The RPA experiment was performed using the primer set with or without DNA from *S. pyogenes,* and the resulting RPA products were examined through electrophoresis. M represents Marker, N represents Negative control, and it serves as a non-template control. (B) The optimal time for RPA detection. The RPA response time was adjusted to 37°C for 5, 10, 15, 20, 25, and 30 min.

To assess the efficiency of the primer sets (Table 1), the genomic DNA from *S. pyogenes* (ATCC 19615) was extracted. In order to determine the optimal time for RPA detection, the RPA experiment was conducted at 37°C for 5, 10, 15, 20, 25, and 30 min. The products were divided on agarose gels and observed under ultraviolet illumination. Figure 3A demonstrated that the size of RPA products using the primer sets was consistent with the anticipated size, and there were no amplification products in the negative control. RPA amplification products can appear in 10, 15, 20, 25, and 30 min (Fig. 3B). Thus, the RPA test can be completed in 10 min.

### Selection of crRNA

**Fig 4:**
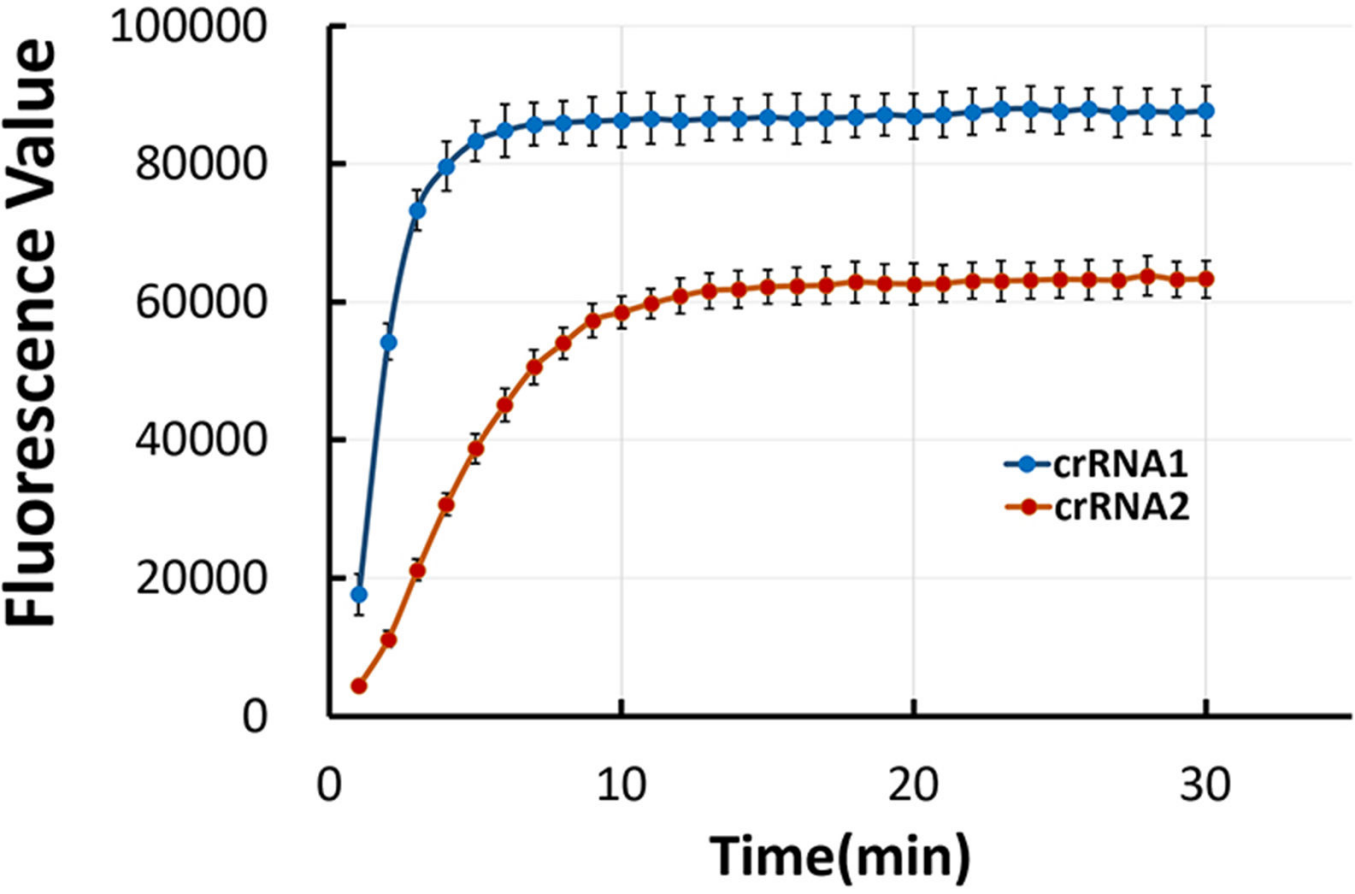
The selection of crRNA Real-time fluorescence signal of two crRNA designed for CRISPR-*S. pyogenes* assay; blue, crRNA 1; orange, crRNA 2. The reaction tube was then placed in the fluorescence PCR instrument. At 37°C, the reaction mixture was incubated for 30 min. The experiment was repeated three times.

By detecting fluorescence signals, we evaluated the optimized crRNA for CRISPR-Cas12a. One microliterRPA product was added to the CRISPR-Cas12a system for fluorescence detection, and the reaction system was incubated at 37°C for 30 min. The results showed that crRNA1 generated the more intense fluorescent signal than crRNA2. Therefore, crRNA1 was selected for the subsequent CRISPR-Cas12a assay (Fig. 4).

The CRISPR-RPA test was developed for *S. pyogenes* by utilizing the CRISPR-Cas12a system along with RPA amplification, which can be easily conducted in a standard laboratory setting. The rapid detection capability of the CRISPR-RPA assay makes it a promising tool for detecting *S. pyogenes* due to its speed and accuracy.

### Sensitivity of the CRISPR- *S. pyogenes* detection assay

After establishing the fluorescence-CRISPR-*S. pyogenes* and LFB-CRISPR- *S. pyogenes* assays, the sensitivities of the detection assays were evaluated. In order to detect the sensitivity of the CRISPR-*S. pyogenes,* the plasmids containing RPA amplified fragment of sdaB (pUC18-sdaB) were extracted. Series concentration pUC18-sdaB DNA was tested both the fluorescence-CRISPR-*S. pyogenes* and the LFB-CRISPR-*S. pyogenes* assays. The minimum concentration at which the CRISPR-*S. pyogenes* tests could identify it was defined as the limit of detection (LoD).

**Fig 5:**
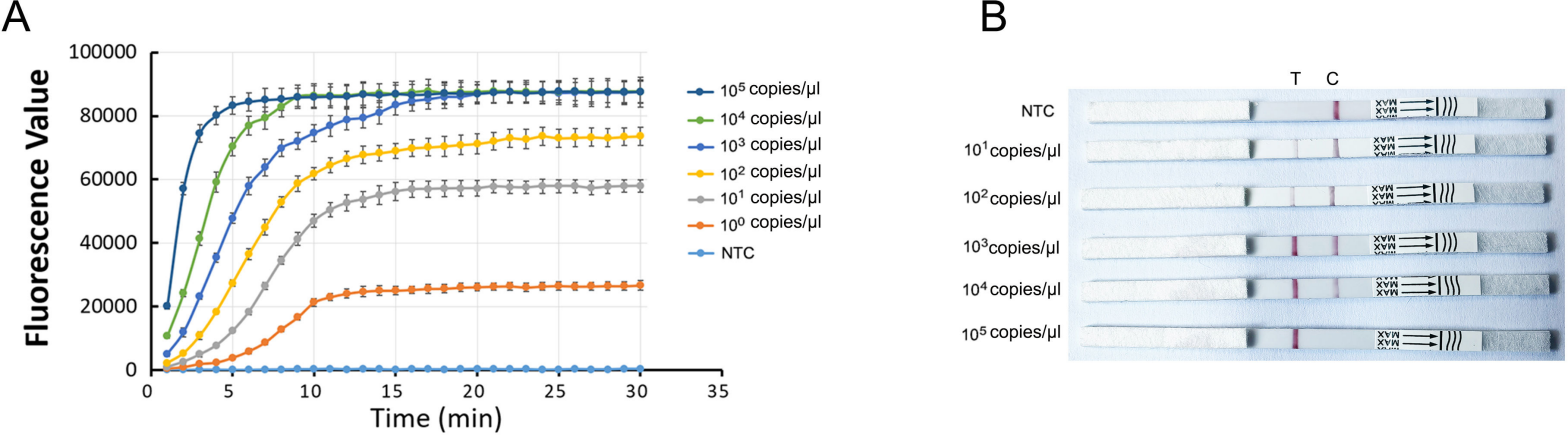
The sensitivity of the CRISPR-detection. (A) The sensitivity of fluorescence-CRISPR-S. *pyogenes* assay. The concentrations are 1 × 10^0^, 1 × 10^1^, 1 × 10^2^, 1 × 10^3^, 1 × 10^4^, 1 × 10^5^ copies/μL, respectively. NTC, non-template control. Error bars represent ±SD, where *n* = 3 independent experiments. (B) The sensitivity of LFB-CRISPR-*S. pyogenes*. The concentrations are 1 × 10^1^, 1 × 10^2^, 1 × 10^3^, 1 × 10^4^, 1 × 10^5^ copies/μL, respectively. NTC, non-template control.

According to Fig. 5A, the fluorescence intensity at the endpoint for a concentration of 1 copy/μL was considerably greater than the fluorescence intensity of the NTC. Thus, the LoD for the fluorescence-CRISPR-*S. pyogenes* assay was 1 copy/μL. The test line of the LFB-CRISPR-*S. pyogenes* assay was observed when the concentration of *S. pyogenes* DNA exceeded 10 copies/μL. The LoD for the LFB-CRISPR-*S. pyogenes* test was determined to be 10 copies/μL (Fig. 5B).

### Specificity of the CRISPR-*S. pyogenes* detection assay

Additionally, the evaluation of the CRISPR-*S. pyogenes* assay’s specificity was conducted. To assess the discriminatory capacity of the CRISPR-*S. pyogenes* test, various common pathogens were used, *Streptococcus pyogenes* (ATCC 19615), *Streptococcus pneumoniae* (ATCC49619), *Streptococcus mitis* (ATCC6249)*, Streptococcus agalactiae* (ATCC13813)*, Streptococcus dysgalactiae* subsp. (ATCC12388)*, Streptococcus anginosus* (ATCC33397)*, Streptococcus bovis* (ATCC35034)*, Streptococcus gallolyticus* (ATCC49147)*, Streptococcus equinus* (JCM7879), *Staphylococcus aureus* (ATCC25923), *Staphylococcus epidermidis* (ATCC12228), *Escherichia coli* (ATCC25922), *Pseudomonas aeruginosa* (ATCC27853), *Listeria monocytogenes* (ATCC19118).

The pathogens’ genomic DNA, including *S. pyogenes,* was extracted following the aforementioned procedures. The RPA procedure was carried out at a temperature of 37°C for 10 min. Fluorescence-CRISPR-*S. pyogenes* and LFB-CRISPR-*S. pyogenes* assays were used for testing the products. Different fluorescence values were observed in *S. pyogenes* and other control bacteria.

**Fig 6:**
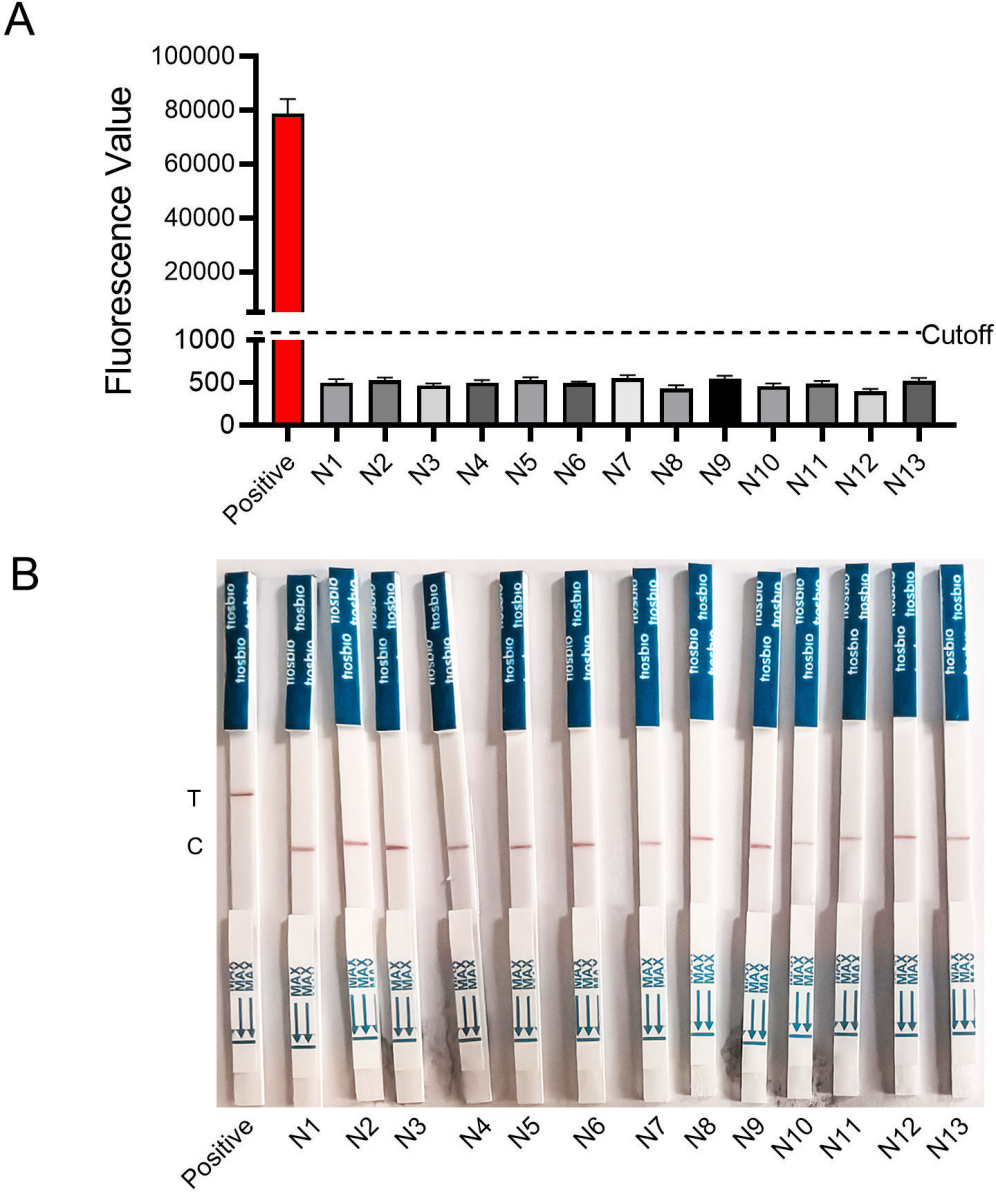
Specificity of CRISPR-*S. pyogenes* assays. (**A**) Results of fluorescence- CRISPR-*S. pyogenes* assay. Error bars represent ±SD, where *n* = 3 independent experiments. (**B**) Results of LFB-CRISPR-*S. pyogenes* assay. Positive: *S. pyogenes*; N1: *Streptococcus pneumoniae*. N2: *Streptococcus mitis*. N3: *Streptococcus agalactiae*. N4: *Streptococcus dysgalactiae* subsp. N5: *Streptococcus anginosus*. N6: *Streptococcus bovis.* N7: *Streptococcus gallolyticus*. N8: *Streptococcus equinus*. N9: *Staphylococcus aureus*. N10: *Staphylococcus epidermidis*. N11: *Escherichia coli*. N12: *Pseudomonas aeruginosa*. N13: *Listeria monocytogenes*.

The results showed that the fluorescence value of *S. pyogenes* was significantly increased compared with other control bacteria (Fig. 6A). According to test lines and control lines, LFB results were observed for *Streptococcus pyogenes* and other control bacteria (Fig. 6B). The fluorescence values of 13 control bacteria were significantly lower than the fluorescent value of *Streptococcus pyogenes*. The LFB results for 13 control bacteria showed red on the control line and *Streptococcus pyogenes* bacteria showed red on the test line. Consequently, the LFB-CRISPR-*S. pyogenes* and fluorescence-CRISPR-*S. pyogenes* tests exhibited specificity in identifying *Streptococcus pyogenes*.

### Validation using clinical samples

**Fig 7:**
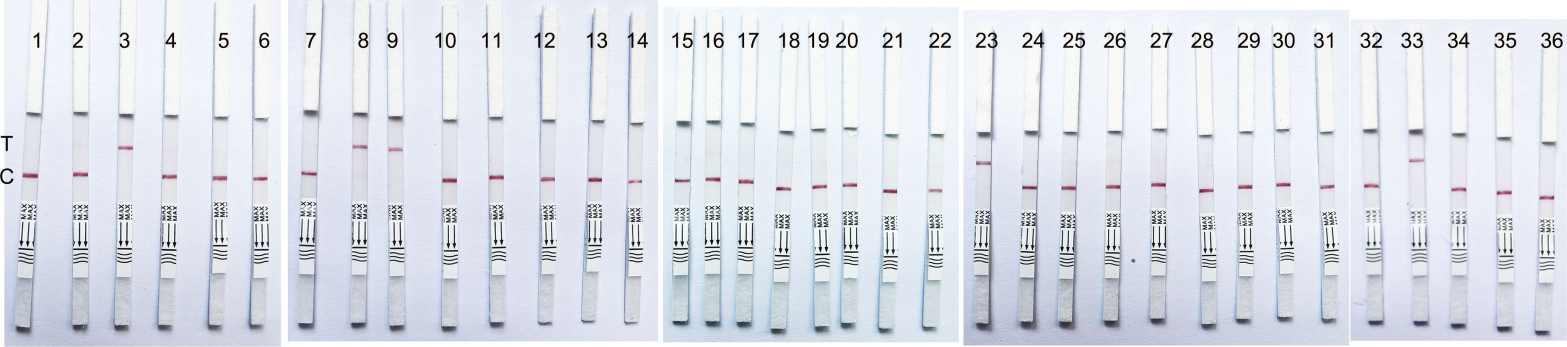
Validation using conjunctival sac secretions samples. The results contained 36 conjunctival sac secretions clinical samples with 31 clinical negative samples and 5 clinical positive samples. All results of these 36 samples are consistent between the LFB-CRISPR-*S. pyogenes* and Real-time PCR.

To validate the clinical performance of the LFB-CRISPR-*S. pyogenes*, we selected 36 conjunctival sac secretions from suspected conjunctivitis patients as clinical samples. The results showed that the five samples were positive according to the LFB-CRISPR-*S. pyogenes* assay results, as shown in Table 5; Fig. 7. The results from Real-time PCR were consistent with the LFB-CRISPR-*S. pyogenes* assay results. There was no statistically significant difference between the two methods (*P* > 0.05). Both assays specifically detected *S. pyogenes* in clinical samples.

**TABLE 5:** Results from conjunctival sac secretions sample testing using the LFB-CRISPR-*S. pyogenes* assay and Real-time PCR

Methods		Real-time PCR			Chi-square value	*P* value
		Positive	Negative	Total		
LFB-CRISPR-*S. pyogenes* assay	Positive	5	0	5	0.000	1.000
	Negative	0	31	31		
	Total	5	31	36		

**Fig 8:**
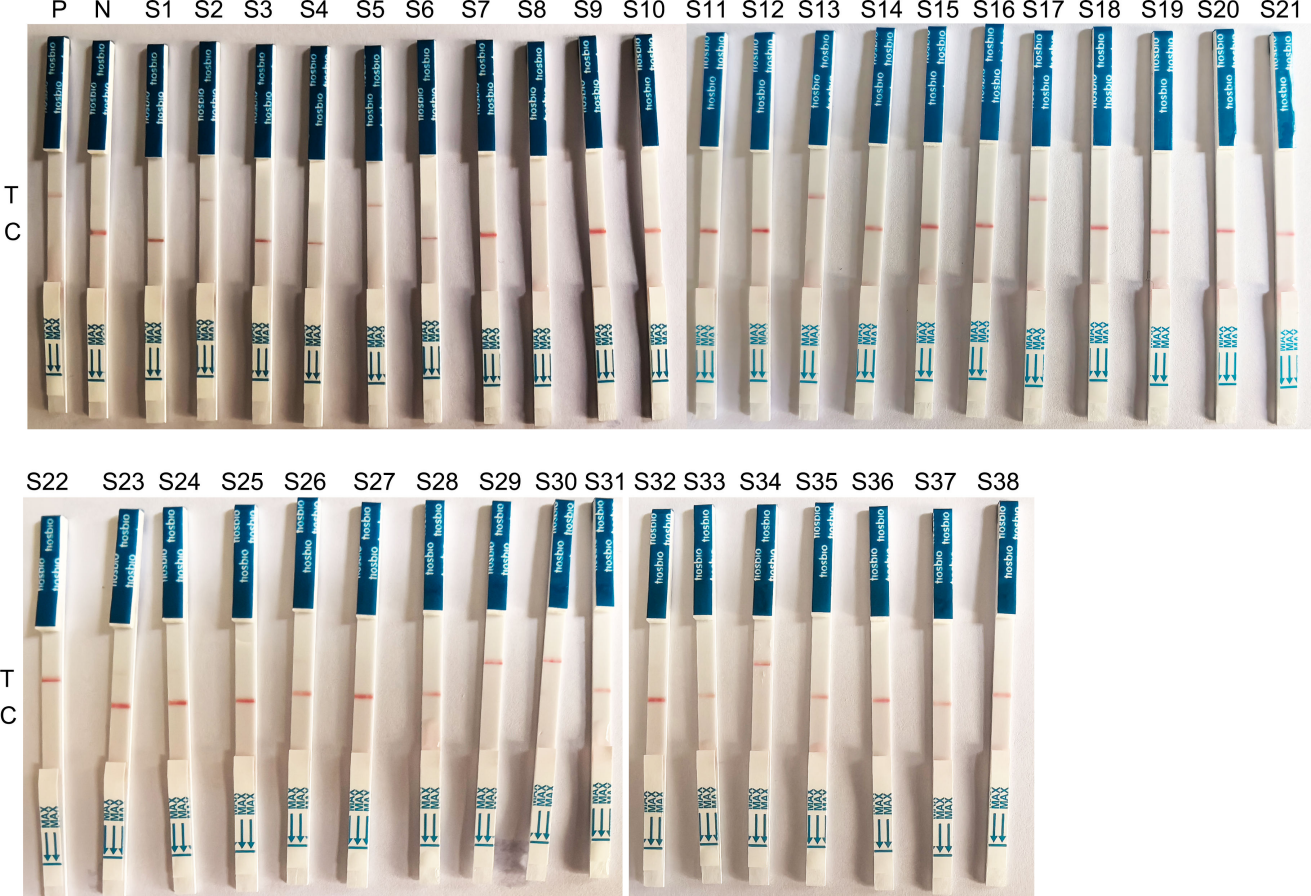
Validation using throat swab samples. The results contained 38 throat swab samples with 29 clinical negative samples and 9 clinical positive samples. All results of these 38 samples are consistent between the LFB-CRISPR-*S. pyogenes* and Real-time PCR.

In order to further validate the LFB-CRISPR-*S. pyogenes*, 38 throat swab samples from pharyngitis patients were also selected. The presence of *S. pyogenes* was confirmed with Real-time PCR. The nine samples were positive according to the LFB-CRISPR-*S. pyogenes assay* results, as shown in Table 6; Fig. 8. The results from Real-time PCR were consistent with the LFB-CRISPR-*S. pyogenes* assay results. Both assays specifically detected *S. pyogenes* in clinical samples. There was no statistically significant difference between the two methods (*P* > 0.05). These results showed that the LFB-CRISPR-*S. pyogenes* exhibited good diagnostic performance.

**TABLE 6:** Results from throat swab samples testing using the LFB-CRISPR-*S. pyogenes* assay and Real-time PCR

Methods		Real-time PCR			Chi-square value	*P* value
		Positive	Negative	Total		
LFB-CRISPR-*S. pyogenes* assay	Positive	9	0	9	0.000	1.000
	Negative	0	29	29		
	Total	9	29	38		

## DISCUSSION

In addition to necrotizing fasciitis and streptococcal toxic shock syndrome, the Group A Streptococcus also leads to serious invasive illnesses (4, 22, 23), while infecting the upper respiratory system, which includes the tonsils and pharynx, and causing postinfection conditions like rheumatic fever and glomerulonephritis. Detection using classical techniques, including culture-based assays, colony morphology, rapid antigen detection, and serology, is time-consuming and can produce equivocal results. There is an urgent need for new, fast, and precise noninvasive techniques to identify *S. pyogenes*. CRISPR-Cas12a has been developed as a new noninvasive and rapid method for detecting *S. pyogenes*.

Nucleic acid detection can be greatly enhanced by the CRISPR-Cas12a system. There have been reports of CRISPR-Cas12a-based detection methods (24–26). To detect *S. pyogenes* infections, we utilized CRISPR-Cas12a technology, which is frequently combined with RPA techniques for nucleic acid detection. A novel test method was developed by combining RPA with CRISPR-Cas12a technology, and we selected the best crRNA for the Cas12a reaction. The sensitivity and specificity of this new method were evaluated. CRISPR-Cas12a recognized target sequences via crRNA resulting in highly specific detection of *S. pyogenes* in clinical specimens without cross-reactivity for other pathogens. When tested using clinical samples, our CRISPR-Cas12a method exhibited good diagnostic performance.

Current *in vitro* diagnostic (IVD) products frequently utilize LFBs for the detection of antibodies or antigens (27–29). In our novel method, LFB was used to detect nucleic acid from *S. pyogenes*. The outcome is visible to the unaided eye and does not require advanced equipment.

A comparison of our CRISPR-Cas12a system with traditional detection methods revealed significant benefits. Traditional methods, which are both time-consuming and laborious, do not allow for species-level identification based on growth characteristics, colony morphology, and biochemical reactions. Gao et al. established a rapid visual method for the detection of *Streptococcus pyogenes* (GAS) based on recombinase polymerase amplification (RPA) and lateral flow strip (LFS). The Cas12a system is not used in the system (30).

In the study, a rapid and precise identification of *S. pyogenes* is achieved within 33 min using a CRISPR-PCR assay that directly detects nucleic acids from clinical samples. The estimated cost per sample is $1–2. This method not only realizes the rapid, sensitive, accurate, and specific detection of target bacteria but also improves the portability and user friendliness of detection and is more suitable for use in areas with a backward economy and lack of experimental resources. This is a convenient and quick method that can be applied in the clinic or at home without the need for any specialized equipment.

But, this method has the potential for cross-contamination. This problem is a little difficult to solve completely, and the best way is to do all the reactions (extraction—amplification —detection) in the same tube (one-pot). However, if one tube is completed, it will be restricted by many factors. For example, RPA and CRISPR reaction conditions are inconsistent, such as ion concentration, pH value, buffer, and so on. So, it is difficult to achieve a real one-pot because of technical limitations. Hu et al. reported the RPA reaction was carried out in the inner tube containing two hydrophobic holes at the bottom. After the completion of the amplification reaction, the reaction solution entered the outer tube containing the prestored Cas13a reagent under the action of centrifugation or shaking. Inner and outer tubes were combined to complete the nucleic acid detection (31).

### Conclusions

A quick and highly responsive method was established in this research to identify *S. pyogenes*. The assay is based on Cas12a/crRNA technology combined with RPA and LFB. In addition to being very rapid, this new Cas12a/crRNA-based assay is highly specific and extremely sensitive. Thus, this new assay is a promising tool for *S. pyogenes* detection. Because of the inclusion of LFB, this test can serve as a point-of-care examination for epidemiological and extensive screening research. However, more specimens are needed to evaluate the specificity and sensitivity of the assay in clinical trials.
